# Intravenous Administration of Achyranthes Bidentata Polypeptides Supports Recovery from Experimental Ischemic Stroke in Vivo

**DOI:** 10.1371/journal.pone.0057055

**Published:** 2013-02-26

**Authors:** Hongmei Shen, Xinmin Wu, Yuzhong Zhu, Hualing Sun

**Affiliations:** Key Laboratory of Neuroregeneration and Institute of Nautical Medicine, Nantong University, Nantong, China; Universidad de Castilla-La Mancha, Spain

## Abstract

**Background:**

*Achyranthes bidentata* Blume (*A. bidentata*) is a commonly prescribed Chinese medicinal herb. *A. bidentata* polypeptides (ABPP) is an active composite constituent, separated from the aqueous extract of *A. bidentata*. Our previous studies have found that ABPP have the neuroprotective function in vitro and in rat middle cerebral artery occlusion (MCAO) model in attenuating the brain infract area induced by focal ischemia-reperfusion. However, the ultimate goal of the stroke treatment is the restoration of behavioral function. Identifying behavioral deficits and therapeutic treatments in animal models of ischemic stroke is essential for potential translational applications.

**Methodology and Principal Findings:**

The effect of ABPP on motor, sensory, and cognitive function in an ischemic stroke model with MCAO was investigated up to day 30. The function recovery monitored by the neurological deficit score, grip test, body asymmetry, beam-balancing task, and the Morris Water Maze. In this study, systemic administration of ABPP by i.v after MCAO decreased the neurological deficit score, ameliorated the forepaw muscle strength, and diminished the motor and sensory asymmetry on 7^th^ and 30^th^ day after MCAO. MCAO has been observed to cause prolonged disturbance of spatial learning and memory in rats using the MWM, and ABPP treatment could improve the spatial learning and memory function, which is impaired by MCAO in rats, on 30^th^ day after MCAO. Then, the viable cells in CA1 region of hippocampus were counted by Nissl staining, and the neuronal cell death were significantly suppressed in the ABPP treated group.

**Conclusion:**

ABPP could improve the recovery of sensory, motor and coordination, and cognitive function in MCAO-induced ischemic rats. And this recovery had a good correlation to the less of neuronal injury in brain.

## Introduction

Ischemic stroke is one of the leading causes for severe chronic morbidity or mortality in humans. Despite intensive research, therapeutic strategies are limited and there is as yet no routine effective, generally accepted, specific and promising approaches for ischemic stroke [1], [2]. Ischemic stroke leads to interruption of brain blood supply and failure of energy-delivering processes, which result in disturbance of essential ionic gradients, excessive neuronal depolarization, and consequently large amounts of excitatory amino acids, mainly glutamate, releases into the extracellular space [3].

Glutamate is the major excitatory neurotransmitter in the mammalian central nervous system (CNS). However, high extracellular glutamate concentration in the CNS is a primary step leading to neuronal injury, specifically referred to as excitotoxicity [4]. It is well known that glutamate can activate three classes of ionotropic postsynaptic receptors, namely AMPA, N-methyl-D-aspartate (NMDA) and kainate receptors, in which NMDA receptors play a key role in mediating excitotoxicity due to their high permeability to Ca^2+^ [5], [6], [7]. Therefore, the excess calcium ion triggered by over-activated NMDA receptors is linearly correlated to neuronal cell damage during the ischemic episode and the reperfusion phase [8], [9], [10], [11]. Developing novel and effective NMDA receptor-based therapy is a very important neuroprotection strategy and offers a huge hope for treating ischemic stroke.

In recent years, there is a growing interest in the treatment of NMDA-induced neurotoxicity with plant-based therapy including traditional Chinese medicine (TCM), for which extensive experience has been accumulated over thousands of years [12], [13]. *Achyranthes bidentata* Blume (A. bidentata), a commonly prescribed traditional Chinese herb, occupies an important position in traditional Chinese stroke therapy owing to the property of promoting the circulation of blood and removing stasis [14]. *A. bidentata* polypeptides (ABPP), an important constituent, which separated from the aqueous extract of *A. bidentata* Blume, could attenuate the NMDA-induced cell apoptosis in cultured hippocampal neurons [15], and an active fraction of ABPP could exert protective effects against serum deprivation induced apoptosis in SH-SY5Y cells [16]. Our previous study has demonstrated that intravenous administration of ABPP can decrease the brain infarct volume and the neurological deficit score at 24 hours after reperfusion in transient MCAO rats [17]. It is well known that characteristics of an ischemic stroke include deficits in motor, sensory, and behavioral function [18], [19]. In the present study, ABPP, the *A. bidentat* derived active constituent, was used to treat the MCAO-induced focal ischemic rats in an attempt to identify its neuroprotective effects.

## Materials and Methods

### Animals and Surgical Procedures

Male SD rats, weighing 200±10 g, were provided by the Experimental Animal Center of Nantong University, Nantong, China. All experimental procedures involving animals were conducted as per institutional animal care guidelines and were approved ethically by the administration committee for experimental animals, Jiangsu Province, China. Transient focal cerebral ischemia was performed as previously described [20]. Briefly, The animals were anesthetized with 2 ml enflurane in an ether jar and maintained with 10% chloral hydrate (400 mg/kg, i.p.), then subjected to MCAO with a silicone-coated nylon suture (Filament-R TY6315, F-24, Shading Biotechnology Ltd. Co., Beijing, China) by surgical operation. After revival from anesthesia, animals were housed back at 25±1°C. Reperfusion was induced at 2 hours after MCAO by filament withdrawal. Sham-operated animals were subjected to an identical surgical procedure except that the suture was not advanced beyond the internal carotid bifurcation. Only the rats, which had undergone MCAO for 2 hours and scored at or above 2 according to the neurological deficit score, were selected for randomization into different groups. A total of 130 rats satisfied the criteria and were included in the study, and 54 rats were excluded because of death after MCAO.

### Physiological Parameters

The rectal temperature of rats was maintained at 37°C throughout the anesthetic and surgical period with a temperature-regulated heating pad. In a parallel group of animals subjected to the same anesthesia and surgery protocol, we measured systolic blood pressure, diastolic blood pressure, mean arterial blood pressure and heart rate of rats with a caudal artery pressure measuring system (Alcott Biotech Co. Ltd, Shanghai, China) at 10 mins before, 15 mins after MCAO and 15 mins after reperfusion after 2 hours focal cerebral ischemia in rats. At the same time, A flexible laser-Doppler probe was glued onto the exposed left parietal skull over the territory of the MCA for continuous monitoring of regional cerebral blood flow, and anther flexible laser-Doppler probe was glued onto the abdominal skin to monitor the homeochronous blood oxygen pressure (PF 5010 to LDPM Unit, PF 5040 to PO_2_, Perimed 5001 Master, Perimed, Sweden).

### Systemic Administration of ABPP

At the first time, 2 hours after MCAO ABPP (0.1 mg/kg, 0.2 mg/kg, and 1.0 mg/kg), dizocilpine (MK801, 1.0 mg/kg) was injected into the tail vein of the awake rats at a speed of 1.0 ml/5 min with a syringe pump. 24 hours after MCAO, half of dose was injected, and a quarter of dose was injected for the next four days. MK801, an antagonist of NMDA receptor, was used for this study because this drug has been studied widely and has demonstrated efficiency in experimental ischemic research [21], [22]. Control animals received the same volume normal saline at the same speed.

### Neurological Deficit Score Test and Infarct Volume Assessment

Behavioral tests were performed blindly, and the room temperature and humidity remained stable for all experiments. All the behavioral experiments were measured without Lund whit noise and bright light. The neurological evaluation was performed based on a five-point scale. The score was 0 for no neurological deficit; 1 for failure to extend left forepaw fully; 2 for circling to the left; 3 for inability to bear weight on the left; 4 for no spontaneous walking with depressed level of consciousness. Only the rats that had undergone MCAO for 2 hr and scored at or above 2 were selected for randomization into different groups.

The infarct volume of the cortex and striatum was assessed with 2,3,5-triphenyltetrazolium chloride (TTC) staining as described previously [23], with modifications. In brief, at 7th days after the induction of ischemia, the rats were anesthetized. The brain was quickly removed and sectioned into consecutive 1-mm-thick coronal slices using a vibratome (VT1000S; Leica Instruments, Jena, Germany). Slices were immediately placed in PBS containing 0.5% TTC at 37°C for 15 min and reversed for another 15-min incubation. TTC-stained slices were washed in PBS three times (each 5 min) and then fixed in buffered 4% formaldehyde solution for 12 hr at 4°C. Afterward, color images of these slices were captured with a video camera (Canon PowerShot S60; Canon, Tokyo, Japan), and the infarct volumes were quantitated using Image Pro Plus 4.1 software (Media Cybernetics, Silver Spring, USA). The percentage of the infarct volume was calculated as [(Vc - Vl)/Vc] × 100, where Vc is the volume of control hemisphere and Vl is the volume of noninfaracted tissue in the lesioned hemisphere.

### Grip Test

The grip test consisted of a string 50 cm in length and 3 cm in diameter, pulled taut between two vertical supports and elevated 2 m above a platform. The animal was placed on the string midway between the supports and measured for 60 seconds at 7^th^ and 30^th^ day after MCAO or sham operation. The evaluated scoring was based on the previous described [24] and modified according to 4-point scale from 0 (best) to 3 (worst). The scoring was as follows: 0 = forelimb and hind legs seize the string firmly and climb freely, 1 = hind legs can seize the string and keep the body balanced,but can not climb, 2 = hind legs can not seize the rope, but the rat did not fall from the string, 3 = fall from the string during the observation period. There were three trials for each rat at 7 days and 30 days after MCAO or sham operation.

### Elevated Body Swing Test

Asymmetric motor behavior was quantitatively analyzed with the use of the elevated body swing test as previously described [25] at 7^th^ and 30^th^ day after MCAO or sham operation. Briefly, the rats were examined for lateral movements/turning when their bodies were suspended by lifting their tails. The frequency of initial turning of head or upper body contralateral to the ischemic side was counted in 20 consecutive trials and was normalized, as follows: % recovery = (lateral turns in 20 trials-10)/10×100%.

### Beam-balancing Task

The rat was placed on an elevated (60 cm) rectangle wooden beam (1 m length, 1.5 cm wide), and its balance ability was observed for 60 s at 7^th^ and 30^th^ day after MCAO or sham operation. The neurological sign was applied and modified according to the previous description of the scoring system [26]. The present scoring system is summarized below: 0 = steady and exhibits normal exploratory behavior, and turn around freely, 1 = steady on the beam, stable equilibrium position within the duration, 2 = shaky posture and movements, but clinging to the wood or hanging the wood with limbs more than the duration, 3 = frequent inability to maintain equilibrium position, and immediately fell from the wood. There were three trials for each rat at 7 and 30 days after MCAO or sham operation.

### Heat Sensitivity Test

Before the heat sensitivity examination, rats were put in plastic boxes and allowed 30 mins for habituation. Heat sensitivity was tested by radiant heat using Hargreaves apparatus (IITC Life Science Inc.) and assessed the forepaw withdrawal latency (FPWL) and the hind paw withdrawal latency (HPWL) at 7^th^ and 30^th^ day after MCAO or sham operation. The radiant heat intensity was adjusted so that basal PWL is between 9–12 seconds, with a cut-off of 18 seconds to prevent tissue damage. The result was expressed as follows: the recovery of forepaw heat sensitivity = the ischemic side FPWL/the contralateral side FPWL and the recovery of hind paw heat sensitivity = the ischemic side HPWL/the contralateral side HPWL.

### Morris Water Maze Task

The Morris water maze (MWM) apparatus consisted of a circular pool (160 cm in diameter, 55 cm in height) filled with water (25±1°C) to a depth of 23 cm. which was located in a quiet room that was decorated with contrast visual cues. The pool was conceptually divided into four quadrants (called zones I, II, III, and IV) and a plexiglas platform (10 cm in diameter) was submerged in zone II such that its surface was 1 cm below the water surface. On the other hand, an automatic tracking system was used to record the swimming pathway of rats, and the escape latency, the swimming distance, and the time in each zone were analyzed by the any-maze 4.82 software. On the 7th and 30th day following the induction of ischemia, the Morris water maze task was performed as described previously [27]. Rats were placed into the maze at different starting zone, facing the pool wall. During the training trial, rats were placed into the water in one of three randomized starting zones, which did not contain the platform. Each rat was given 90 seconds to find the invisible platform. If the rat did not succeed within 90 seconds, it was guided onto the platform with a stick. The rat was then allowed to stay on the platform for 30 seconds to familiarize itself with the location of the platform relative to the visual clues. Each rat was given four trials daily for four consecutive days, with an inter-trial interval of 2 hours. They then received a probe test in which the platform was removed from the pool. During the probe test, the rats swam freely for 90 seconds and their behavior was recorded.

### Nissl Staining

After the Morris water maze testing, the rats were anesthetized with 10% chloral hydrate (400 mg/kg, i.p.) and perfused with 250 ml saline and subsequently with 4% paraformaldehyde in 0.1 mol/L PBS (pH 7.4) for 30 mins. Rat brains were removed and post-fixed for 24 hours in the same fixative solution at 4°C, and then transferred to 30% sucrose for 3 days at 4°C. Coronal frozen sections (15 µm thick) through hippocampus were prepared and stained with cresyl violet (Nissl) for evaluation of general neuronal morphology [28], [29]. The neuronal density in the CA1 region was calculated as described previously [30], [31], [32]. The data were represented as the number of neurons per mm.

### Statistical Analysis

All data, unless otherwise indicated (e.g., in figure legends), are presented as means ± SEM. All statistical analysis were performed using the Statistical Package for Social Science (SPSS version 16.0), and statistical differences between groups were analyzed by one-way analysis of variance (ANOVA) followed by subsequent Turkey’s tests. A non-parametric Kruskall-Wallis test was performed on all neurological scoring data, which is the data of neurological deficit score, grip test, and beam-balancing task. Differences were considered statistically significant at *P*<0.05.

## Results

### ABPP Affected the Mortality and the Infarct Volume Following Ischemia

In the experiments, the effects of normal saline (NS), ABPP (1.0 mg/kg), MK801 (1.0 mg/kg) on systolic blood pressure, diastolic blood pressure, mean arterial blood pressure, heart rate, regional cerebral blood flow and blood oxygen pressure were detected. Results demonstrated that the physiological parameters of the rats were unvaried following intravenous injection of NS, ABPP, and MK801 for 30 mins and 60 mins respectively (Figure S1). In the parallel group, the flow of brain blood was declined significantly in rats subjected to the surgery protocol, while other physiological parameters displayed no difference (Figure S2).

**Figure 1:**
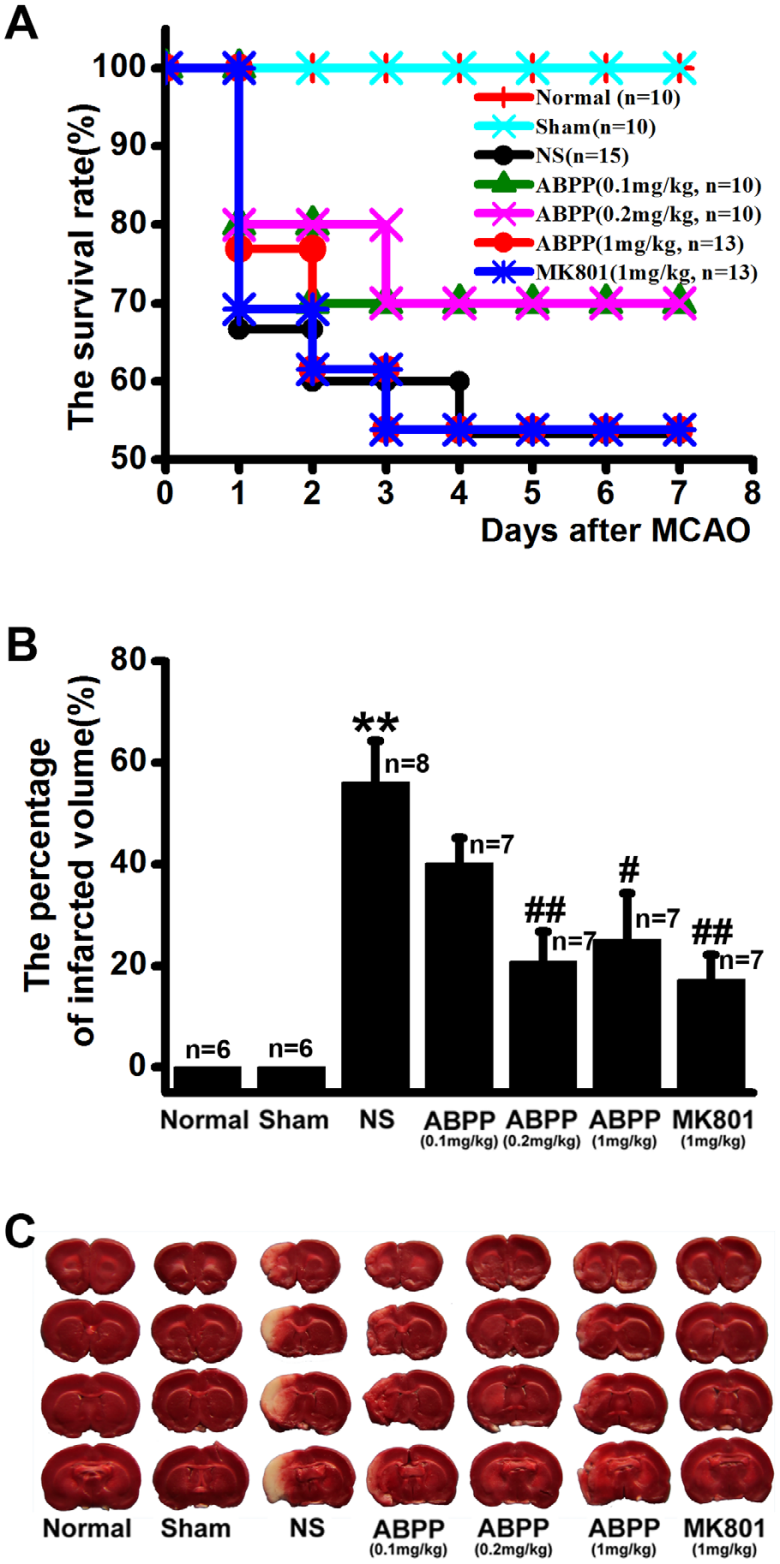
Effects of ABPP on the mortality and the brain infarct volume. Treatment with ABPP did not lower the mortality of ischemic rats (A, *P*>0.05, chi-square test), but did significantly decrease the brain infarct volumes at 7^th^ day after MCAO (B). Also shown is the representative TTC staining for the different groups (C). Data are expressed as means ± SEM; **P<0.01, compared to the Sham group; #*P*<0.05, ##*P*<0.01, compared to the NS group.

To determine neuroprotection of ABPP from cerebral ischemia-reperfusion insults, animals were subjected to 2 hr of ischemia. ABPP (0.1 mg/kg, 0.2 mg/kg, and 1 mg/kg) or MK-801 (1 mg/kg) was systemically administered i.v. from the start of reperfusion to ensure maximum protection. The rat mortality, and the brain infarct volume in saline control group were all significantly increased compared with those in the sham-operated group (Figure 1A). Although the rat mortality was not decreased in ABPP or MK-801 group compared with the saline control group, the percentage of brain infarct volume was 20.8% ±8.9%, 25.1% ±9.2%, and 17.2% ±5.0% in ABPP (0.2 mg/kg, 1 mg/kg) and MK-801 (1 mg/kg) groups, respectively, significantly lower than the volume in the saline control group (56.2%±8.1%; Figure 1B). In other words, post-ischemia administration of ABPP at 0.2 mg/kg, 1 mg/kg, or MK-801 at 1 mg/kg resulted in about a 35%, 30%, or 40% decrease in the brain infarct volume, respectively, compared with the saline control group.

### ABPP Reversed the Motor Impairments Following Ischemia

**Figure 2:**
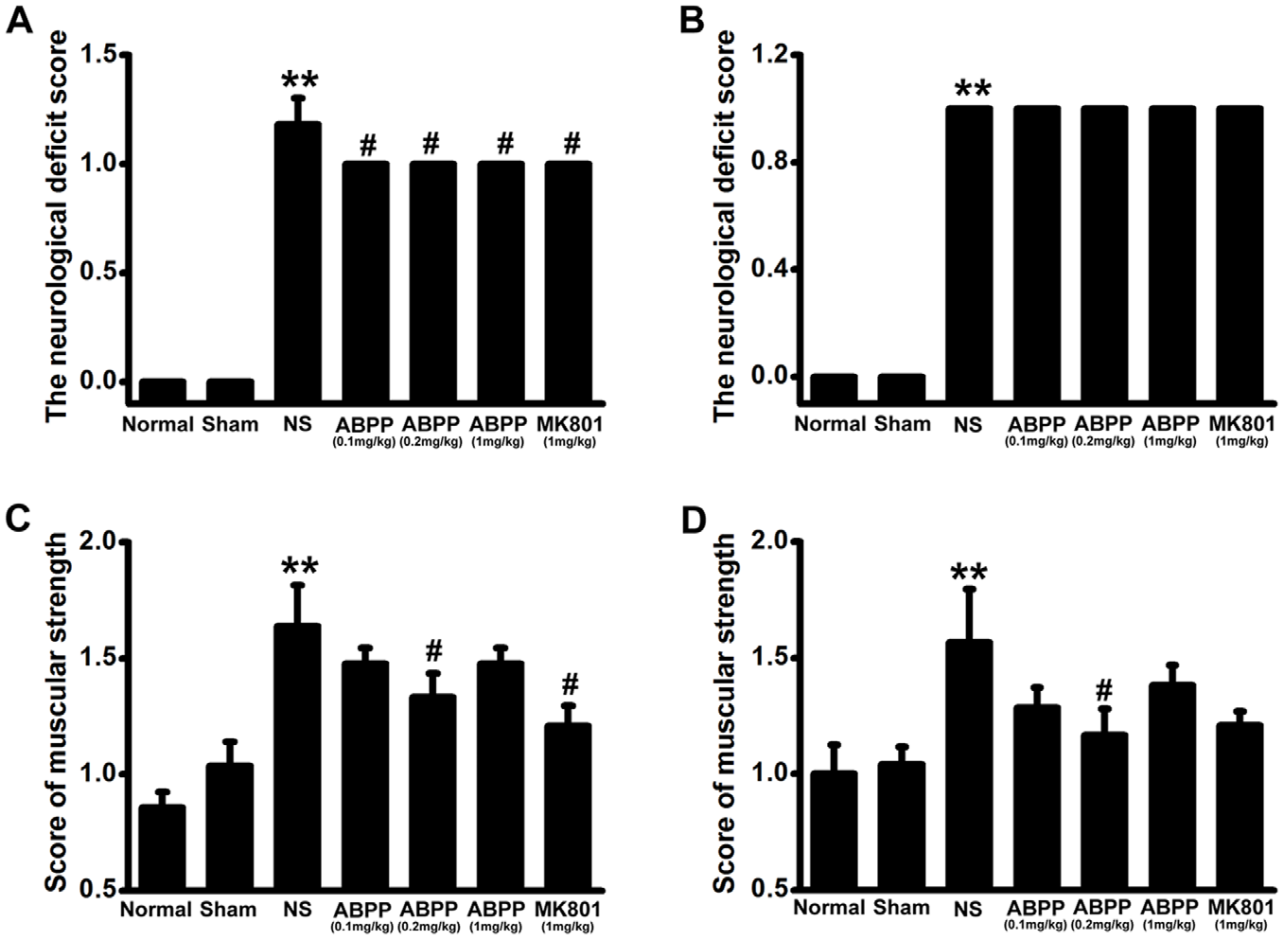
Effect of ABPP on the neurological deficit score and the muscular strength. Treatment with ABPP reduced the neurological deficit score at 7^th^ day after MCAO (A), but did not reduce the score at 30^th^ day (B). However, treatment with ABPP reduced the score of muscular strength at 7^th^ (C) and 30^th^ day (D). Data are expressed as means ± SEM (n = 8); **P<0.01, compared to the Sham group; #*P*<0.05, compared to the NS group.

The neurological deficit score in saline control treated MCAO group was significantly increased compared with those in the sham-operated group (*P*<0.001, Figure 2A, 2B). In rats treated with ABPP (0.1 mg/kg; 0.2 mg/kg; 1 mg/kg) or MK-801 (1 mg/kg), the neurological deficit score was lower at 7 days after the induction of ischemia compared to the saline control treated MCAO group (*P*<0.05, Figure 2A ). Compared to the sham-operated group, the muscle strength measured by the grip test, significantly decreased in the saline control treated MCAO rats, (*P*<0.0001, Figure 2C, 2D). Following treatment with ABPP (0.2 mg/kg), the rats showed a low score of muscle strength at 7 and 30 days after MCAO (*P*<0.05, Figure 2C, 2D).

### ABPP Reversed the Motor Coordination Impairments Following Ischemia

**Figure 3:**
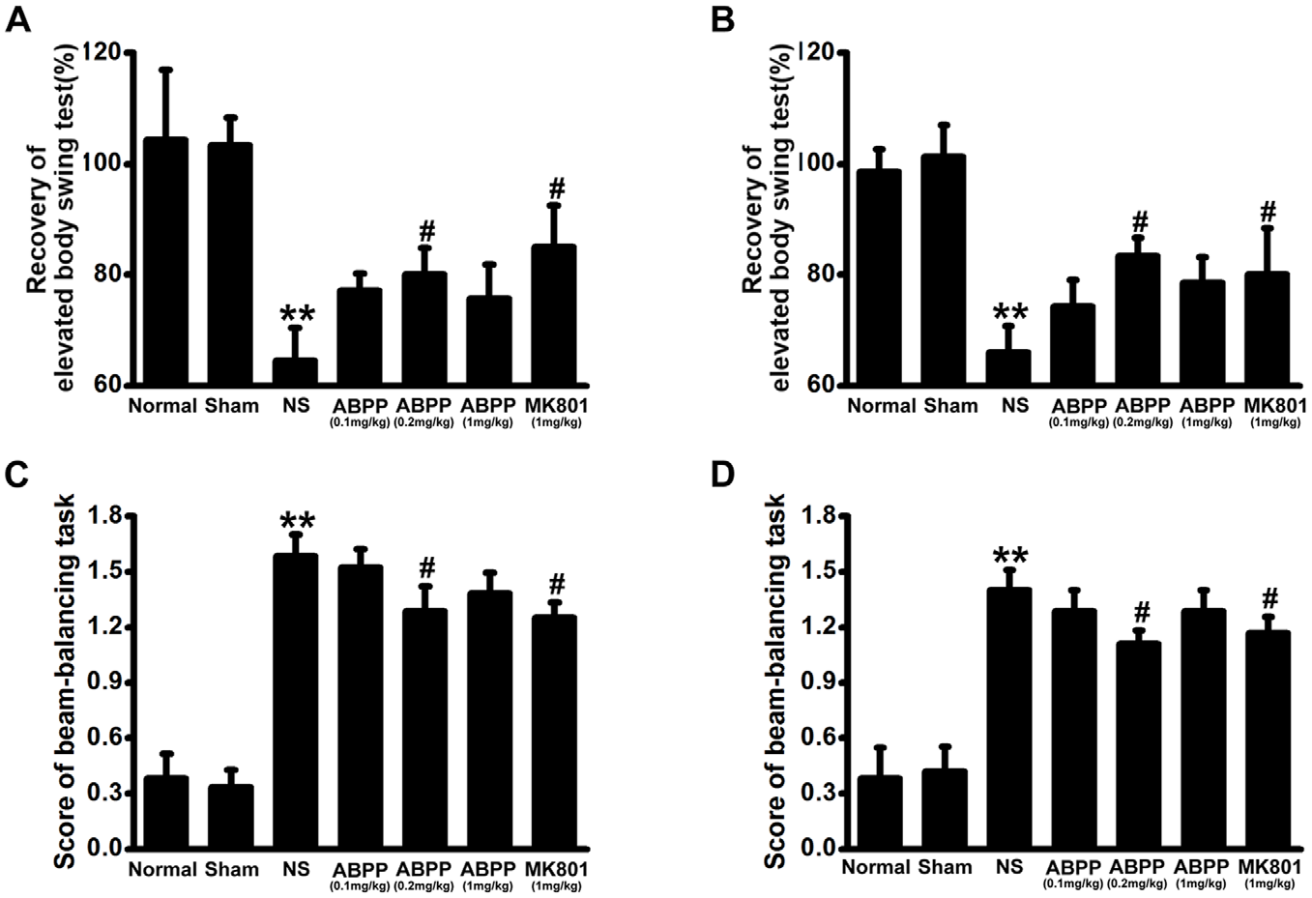
Effect of ABPP on the elevated body swing test and the score of beam-balancing task. Systemic administration of ABPP improved asymmetric motor behavior at 7^th^ (A) and 30^th^ day (B) after MCAO. Treatment with ABPP reduced the beam-balancing task score at 7^th^ (C) and 30^th^ day (D) too. Data are expressed as means ± SEM (n = 8); **P<0.01, compared to the Sham group; #*P*<0.05, compared to the NS group.

An elevated body swing test was used to evaluate asymmetric motor behavior at 7 and 30 days after MCAO. In saline control treated MCAO group, rats developed significant motor asymmetry at 7 and 30 days (*P*<0.01, Figure 3A, 3B). Intravenous administration of a high dose (1 mg/kg) of ABPP was not able to prevent the development of asymmetry (Figure 3A, 3B, *P*>0.05), while a dose of 0.2 mg/kg ABPP showed the prevention at 7 and 30 days (*P*<0.05, Figure 3A, 3B). The beam balancing test can detect the fine motor coordination [33], [34], [35]. In saline control treated MCAO group, rats displayed a high score of beam balancing task (Figure 3C, 3D, *P* *<*0.01). Following treatment with ABPP, the rats showed a lower score at 7 and 30 days after MCAO (Figure 3C, 3D, *P*<0.05).

### ABPP Reversed the Heat Sensitivity Impairments Following Ischemia

**Figure 4:**
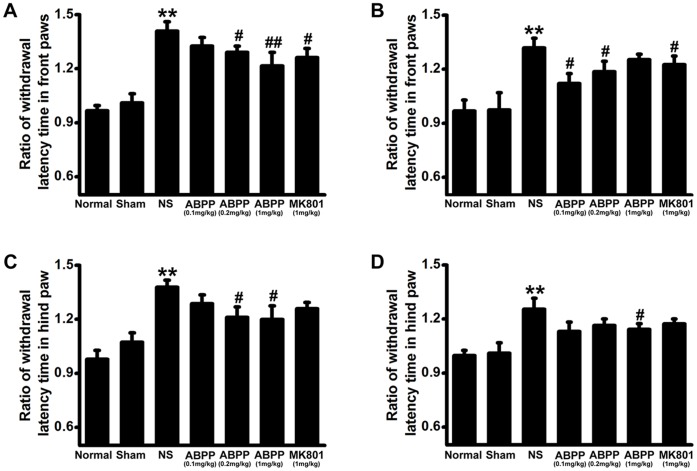
Effect of ABPP on the withdrawal latency time in front paw and in hind paw. Systemic administration of ABPP reduced the ratio of withdrawal latency time in the front paw at 7^th^ (A) and 30^th^ day (B) after MCAO. Treatment with ABPP reduced the ratio of withdrawal latency time in the hind paw at 7^th^ (C) and 30^th^ day (D). Data are expressed as means ± SEM (n = 8); **P<0.01, compared to the Sham group; #*P*<0.05, compared to the NS group.

Heat sensitivity examination was used to evaluate the recovery of sensation on 7 and 30 days after MCAO. In saline control treated MCAO group, the heat sensitivity of the ischemic side was lower than that of the contralateral side in the FPWL and HPWL of rats at 7 and 30 days after MCAO (*P*<0.01, Figure 4). Intravenous administration of ABPP was capable of increasing the heat sensitivity (*P*<0.05, Figure 4). Thus ABPP can improve the recovery of the rats’ heat sensitivity.

### ABPP Reversed the Spatial Learning and Memory Impairments Following Ischemia

**Figure 5:**
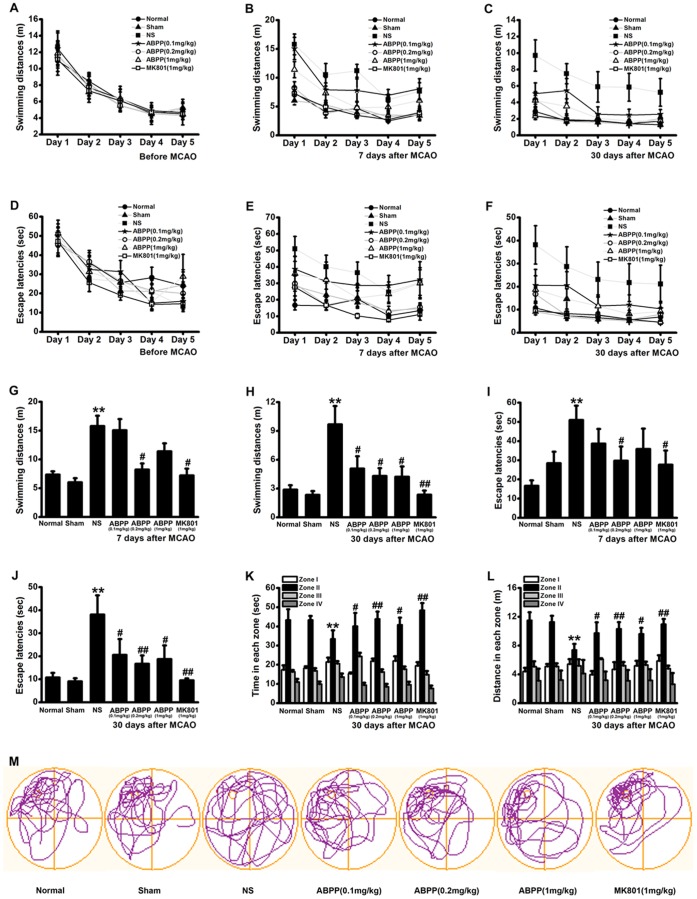
Effect of ABPP on the spatial learning and memory impairments. Swimming distances observed during 5 consecutive days of training on the MWM before MCAO, at 7^th^ and 30^th^ day after MCAO (A, B, C). Escape latencies seen during 5 consecutive days of training on the MWM before MCAO, at 7^th^ and 30^th^ day after MCAO (D, E, and F). Swimming distances observed at 5^th^ day of the training (G, H). At the same time, escape latencies detected (I, J). Time spent and distances swam in the target quadrant (zone II) during the MWM probe test at 30^th^ day (K and L). Swimming paths taken by a representative rat from each group during the probe test (M). Data are expressed as means ± SEM (n = 8); **P<0.01, compared to the Sham group; #*P*<0.05, ##*P*<0.01, compared to the NS group.

Separate groups of rats were suffered to MCAO or sham MCAO surgery, followed 6 days intervention, as described in the Materials and Methods. To investigate the effect of ABPP on spatial learning and memory following MCAO, rats were trained and tested on the MWM before MCAO and on 7 and 30 days after MCAO. Before MCAO, the rats of each group exhibited no difference in spatial learning abilities as indicated by both escape latency and distance traveled prior to locating the platform (Figure 5A, 5D). The spatial learning abilities also were detected on 7 and 30 days following MCAO. ABPP treated groups showed significantly lower escape latency time and total distance travelled than the saline treated group (Figure 5B, 5C, 5E–5J). These findings indicated that the spatial learning impairments displayed following brain ischemia were reversed by post-ischemic ABPP treatment. Spatial memory was also measured in a probe test conducted after the final training trial on 35^th^ day after MCAO. During this test, the platform was removed and time spent in each quadrant of the MWM was recorded. Consistent with the above findings, the saline control treated MCAO group spent much less time and traveled less distance in the target quadrant (zone II) than the sham-operated group did, while the ABPP treated group spent significantly more time and traveled more distance in the target quadrant than the control (*P*<0.01, Figure 5K–5M). These findings indicated that spatial memory impairments showed following brain ischemia were reversed by post-ischemic ABPP treatment. After the MWM tests, the rats were sacrificed for Nissl staining.

### ABPP Inhibited the Neuronal Loss Following MCAO

**Figure 6:**
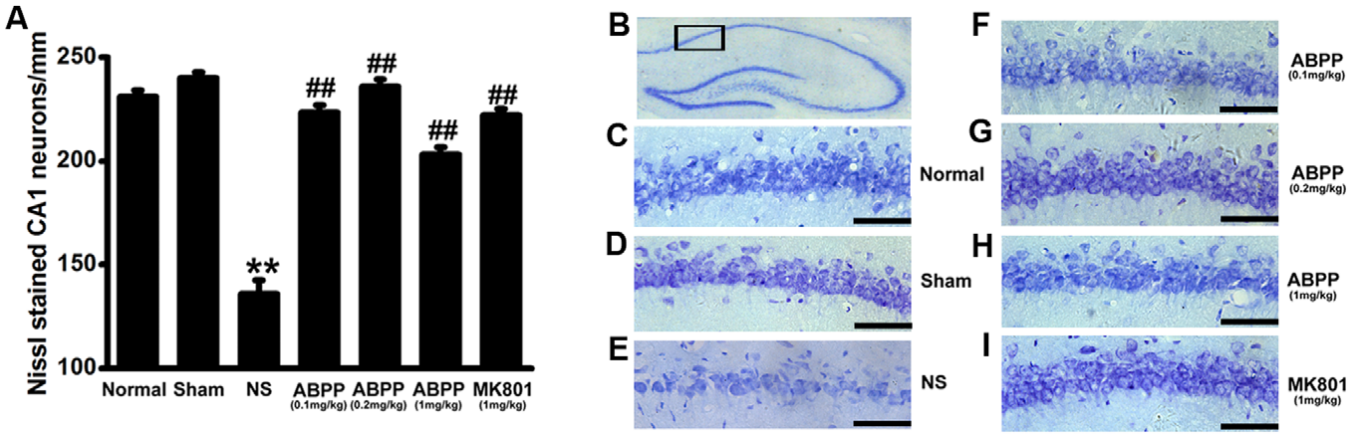
Effect of ABPP on the neuronal density in the CA1 region of hippocampus. Systemic administration of ABPP inhibited the decrease of neuron density induced by MCAO at 30^th^ day after MCAO (A). Schematic depicting hippocampal subregions of interest (B). Neuronal density taken by a representative rat from each group at 30^th^ day after MCAO (C-I; bar, 50 µm). Data are expressed as means ± SEM (n = 8); **P<0.01, compared to the Sham group; ##*P*<0.01, compared to the NS group.

Normal neurons contained Nissl substance in the cytoplasm, loose chromatin, and prominent nucleoli. In contrast, the damaged neurons were identified by the loss of Nissl substance. Neuronal injury was quantified by calculating neuronal density in the CA1 region as described previously [32]. In the saline control MCAO treated group, the number of Nissl-stained neurons was greatly reduced compared with the sham-operated group, while treatment with ABPP resulted in a significant increase in the Nissl positive neurons compared with the control (*P*<0.05, Figure 6).

## Discussion

In the present study, transient MCAO was used as a model to induce ischemia and reperfusion insults [36] for examining the neuroprotective effects of ABPP. Here, our data have demonstrated that the intravenous administration of ABPP ameliorated sensation, motor, coordination, and cognitive impairment and was accompanied by the reduction of cortex and striatum infarct volume and neuronal cell death in CA1 area of hippocampus. These improvements in ABPP treated ischemic stroke animals indicated the therapeutic potential of ABPP, characterized by attenuation of MCAO-induced behavioral and histological deficit.

Stroke is the second most common cause of death worldwide and ischemic stroke in humans accounts for approximately 80% of all strokes. Ischemic stroke mostly results from thrombotic or embolic occlusion that decreases or suppresses the flow of blood in the middle cerebral artery (MCA), one of the main arteries supplying blood to the brain [37]. Experimental focal cerebral ischemia models have been developed to mimic human stroke and serve as an indispensable tool in the stroke research field [38]. Among experimental ischemic stroke models, the transient MCAO in rats is the most frequently used model to analyze the mechanisms and to study potential treatments. In this study, the transient MCAO model was used by inserting a silicone-coated nylon into the internal carotid artery and advancing until the brain blood flow decreases significantly (Figure S2), and 2 hours later it allows reperfusion by retracting the suture and the brain blood flow recovery (Figure S2). Experimental evidence supports the concept that establishing reperfusion alone is not enough to cease ischemic injury and each step of the ischemic cascade may be a genuine target for therapeutic intervention [38]. A large number of potentially neuroprotective agents directed at different harmful mechanisms in the ischemic cascade have been investigated in experimental animal stroke studies. However, no single neuroprotective agent has successfully survived human clinical trials [39]. It is appealing to develop combination therapies for ischemia, but many drug combinations are fraught with difficulty in designing the blend proportion [39], [40]. In this regard, the composite extracts from natural plants might be effective for ischemic stroke.

Achyranthes bidentata polypeptides (ABPP), an important and composite constituent from the aqueous extract of Achyranthes bidentata Blume, might target different ischemic cascades, while a single neuroprotective agent, for example DP-B99 (a metal ion chelator), directed at one aspect in these cascades. Our previous study has demonstrated that intravenous administration of ABPP reduced the volume of brain infarction and improved neurological outcome at 24 hours after MCAO [17]. In an in vitro experimental model, ABPP could prevent the NMDA-induced neuronal apoptosis through inhibition of the excess Ca^2+^ accumulation, reactive oxygen species production, and Bax expression [15], [17]. Furthermore, ABPP could exert protective effects against the serum deprivation induced apoptosis via PI3K/AKT/Gsk3β pathway [16]. Thus, ABPP could target different cascades in ischemic stroke and might be a potentially neuroprotective agent. In order to determine the efficacy of potentially therapeutic agent, the characterization of long-term functional recovery is critical in experimental strokes [38]. The present study focused on the effects of ABPP treatment on neurological deficits after brain ischemia.

The neurological deficit score is one of the most common neurological scales in animal studies of stroke. Systemic administration of ABPP decreased the neurological deficit score at the 7^th^ day after MCAO, but there was no difference between ABPP group and MK801 group (Figure 3A). At 30^th^ day after MCAO, the neurological deficit score of each MCAO treatment group is 1 (Figure 3B), which indicated failure to extend left forepaw fully. In order to evaluate motor deficits effectively, the grip test was used to measure the maximal muscle strength of forelimbs and combined forelimbs and hind limbs. Our results displayed that treatment with ABPP ameliorated the muscle strength (Figure 3C, 3D). It is well accepted that an ischemic stroke can cause limpness and lack of sensation on one side of the body. Therefore, the behavioral tests were designed to examine the differences in function between the intact and impaired side of the body [42]. The body asymmetry test and the beam balancing function were used to detect the motor asymmetry, while the withdrawal latency time was to assess the sensory asymmetry. The data indicated that ABPP decreased the differences in motor and sensory function made by the unilateral MCAO in rats (Figure 4, 5). Since learning and memory impairments are also common after stroke, cognitive testing is a crucial component in understanding the full scope of deficits. The MWM was used to evaluate spatial learning and memory impairment [42], [43], [44]. In accordance with previous studies [41], [42], [43], MCAO caused the prolonged disturbance of spatial learning and memory (Figure 6). In addition, our data indicated that ABPP was able to improve functions of spatial learning and memory of focal cerebral ischemia rats. The amelioration of the cognitive function might be linked to the neuronal cell density in hippocampus.

In conclusion, treatment with ABPP, the constituents separated from the aqueous extract of *A.bidentata*, could improve the recovery of sensory, motor and coordination, and cognitive functions in experimental MCAO induced ischemic rats. This recovery had a good correlation to the brain infracted area and the neuronal cell density in brain.

## Supporting Information

Figure S1 **Effect of ABPP on physiologic parameters.** The parameters were detected at 10 mins before treatment with normal saline (NS), ABPP (1 mg/kg) or MK801 (1 mg/kg) in rats. NS, ABPP or MK801 did not change the physiologic parameters: the local cerebral blood flow (A), transcutaneous oxygen pressure (B), heart rate (C), systolic blood pressure (D), diastolic blood pressure (E), mean arterial blood pressure (F). Data are expressed as means ± SEM (n = 6).
(TIF)

Figure S2 **Effect MCAO on the physiologic parameters.** The parameters were detected at 10 mins before ischemia induced by MCAO, 15 mins after MCAO, and 15 mins after reperfusion after 2 hours of focal cerebral ischemia in rats. MCAO led to decrease the local cerebral blood flow (A), but it did not change the other physiologic parameters: transcutaneous oxygen pressure (B), heart rate (C), systolic blood pressure (D), diastolic blood pressure (E), mean arterial blood pressure (F). Data are expressed as means ± SEM (n = 8); ** *P*<0.01 compared to before ischemia.
(TIF)
