# Supplementary figures and images for: Intravenous Administration of Achyranthes Bidentata Polypeptides Supports Recovery from Experimental Ischemic Stroke in Vivo
Source: PLoS One. 2013 Feb 26;8(2):e57055. doi: 10.1371/journal.pone.0057055 (PMC3582638; doi:10.1371/journal.pone.0057055)

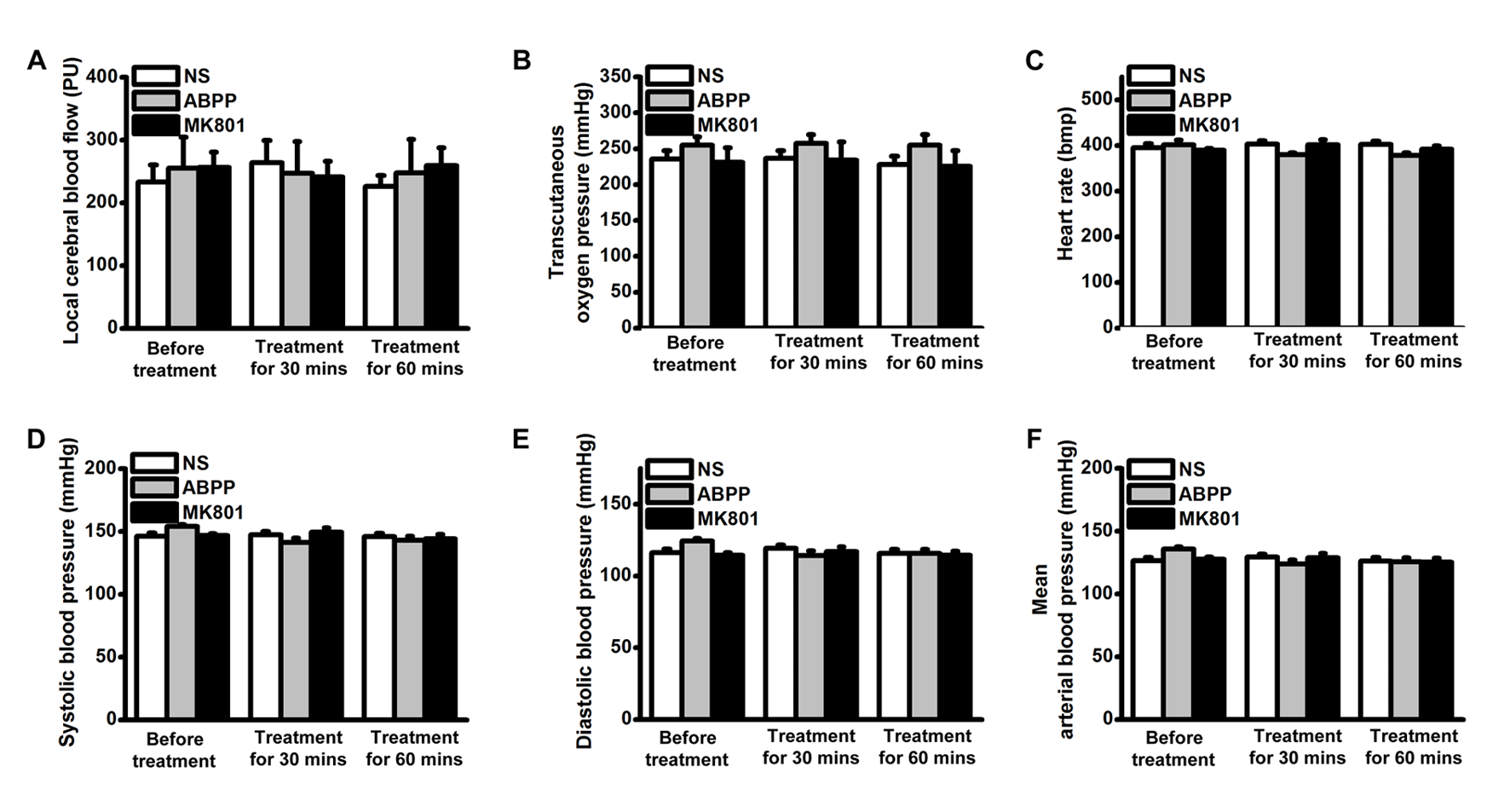

Supplement: Figure S1 — Effect of ABPP on physiologic parameters. The parameters were detected at 10 mins before treatment with normal saline (NS), ABPP (1 mg/kg) or MK801 (1 mg/kg) in rats. NS, ABPP or MK801 did not change the physiologic parameters: the local cerebral blood flow (A), transcutaneous oxygen pressure (B), heart rate (C), systolic blood pressure (D), diastolic blood pressure (E), mean arterial blood pressure (F). Data are expressed as means ± SEM (n = 6). (TIF) [file pone.0057055.s001.tif]

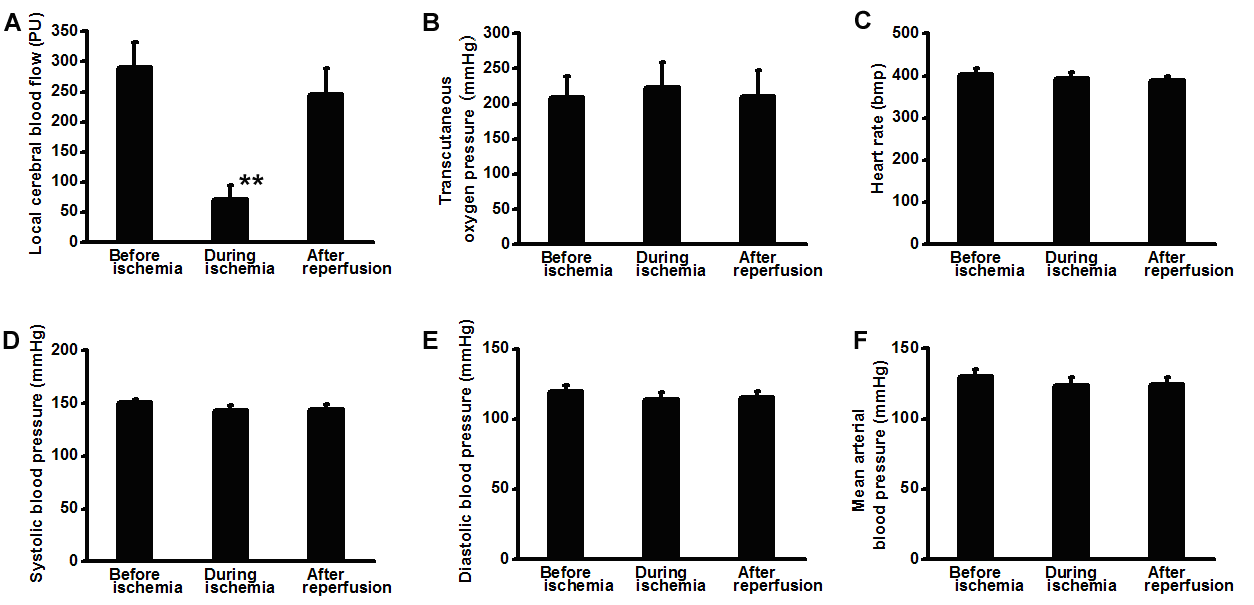

Supplement: Figure S2 — Effect MCAO on the physiologic parameters. The parameters were detected at 10 mins before ischemia induced by MCAO, 15 mins after MCAO, and 15 mins after reperfusion after 2 hours of focal cerebral ischemia in rats. MCAO led to decrease the local cerebral blood flow (A), but it did not change the other physiologic parameters: transcutaneous oxygen pressure (B), heart rate (C), systolic blood pressure (D), diastolic blood pressure (E), mean arterial blood pressure (F). Data are expressed as means ± SEM (n = 8); ** P<0.01 compared to before ischemia. (TIF) [file pone.0057055.s002.tif]
